# Aberrant expression of maternal Plk1 and Dctn3 results in the developmental failure of human *in-vivo*- and *in-vitro*-matured oocytes

**DOI:** 10.1038/srep08192

**Published:** 2015-02-03

**Authors:** Yong Fan, Hong-Cui Zhao, Jianqiao Liu, Tao Tan, Ting Ding, Rong Li, Yue Zhao, Jie Yan, Xiaofang Sun, Yang Yu, Jie Qiao

**Affiliations:** 1 Key Laboratory for Major Obstetric Diseases of Guangdong Province, The Third Affiliated Hospital of Guangzhou Medical University, Guangzhou, 510150, China; 2 Center of Reproductive Medicine, Department of Obstetrics and Gynecology, Peking University Third Hospital, Beijing, 100191, China; 3 Key Laboratory of Assisted Reproduction, Ministry of Education, Beijing, 100191, China; 4 Beijing Key Laboratory of Reproductive Endocrinology and Assisted Reproductive Technology, Beijing, 100191, China; 5 Yunnan Key Laboratory of Primate Biomedical Research and Kunming Biomed International and National Engineering Research Center of Biomedicine and Animal Science, Kunming, 650500, China

## Abstract

Fertilisation is the first step in embryonic development, and dynamic changes of key genes may potentially improve assisted reproduction techniques efficiency during this process. Here, we analysed genes that were differentially expressed between oocytes and zygotes and focused on cytokinesis-related genes. Plk1 and Dctn3 were identified as showing dramatic changes in expression during fertilisation and were suggested to play a key role in inducing aneuploidy in zygotes and 8-cell embryos. Moreover, we found that maternal Plk1 and Dctn3 were expressed at lower levels in *in vitro* matured oocytes, which may have contributed to the high ratio of resulting embryos with abnormal Plk1 and Dctn3 expression levels, thereby reducing the developmental competence of the resulting embryos. Furthermore, the overexpression of Dctn3 can silence Plk1 expression, which suggests a potential regulation mechanism. In conclusion, our present study showed that aberrant expression of Plk1 and Dctn3 increases embryo aneuploidy and developmental failure, particularly in *in vitro* matured oocytes. Our results facilitate a better understanding of the effects of oocyte maternal gene expression on embryonic development and can be used to improve the outcome of assisted reproduction techniques.

Assisted reproductive technology (ART) has remained one of the best methods for infertile couples to obtain offspring since the first baby conceived by *in vitro* fertilisation technology was born in 1978^1^. The pregnancy rate of patients in ART cycles has reached 40%, but the success rate of full-term development remains unacceptable. Phenomena including implantation failure, abortion and premature delivery occur more frequently in ART patients. These failures in ART are mainly attributed to embryonic factors, as they play key roles in the failure or success of both pregnancy and delivery.

Oocyte *in vitro* maturation (IVM) has been successful in clinical settings since 1991^2^ and offers some advantages compared with traditional controlled ovarian hyperstimulation in ART cycles, including reduced odds of ovarian hyperstimulation syndrome, much improved outcome for patients with ovarian dysfunction, and an alternative method for patients who are sensitive to gonadotropins or suffered from other diseases that make superovulation impossible. Five thousand babies have been born with the assistance of IVM technology^3^; however, its poor outcome, attributed to the increased number of low-quality embryos resulting from asynchronised maturation of nuclear and cytoplasmic components, prevents it from being widely applied in the clinic. Therefore, identifying the genes that regulate IVM oocyte maturation is key to improving the quality of resulting embryos.

To improve the outcome of ART, clinicians select which embryos are suitable to be transferred according to different embryo grading criteria based on morphology^4^. However, some studies revealed conflicting results regarding these embryo screening criteria, and importantly, the results in these studies indicated that aneuploidy was observed even in ART embryos with normal morphologies^5^,^6^.

Aneuploidy can contribute to the failure of embryonic development in pre- and post-implantation stages, especially for embryos from human IVM oocytes^7^,^8^. Aneuploidy is relatively common at later developmental stages, affecting at least 4–5% of all clinical pregnancies, the vast majority of which end in miscarriage^9^. In the fertilisation process, sperm with haploid nuclear genomes enter the oocyte and integrate with the haploid nuclear genome of the oocyte, forming a zygote with a diploid nuclear genome. However, some treatments, including IVM^7^ and micromanipulation^10^, lead to aneuploidy when used as part of ART^11^. Compared with sperm, the oocyte spindle-chromosome complex is more easily perturbed based on physical or chemical predisposition. Aneuploid nuclear genomes may result in the aberrant development of fertilised embryos, particularly for women over 35 years old^11^. Therefore, pre-implantation genetic screening (PGS) and pre-implantation genetic diagnosis (PGD) technologies have been applied in the clinic^12^. Some embryos are discarded in this process, and patients face a potential dilemma that no embryos will be transferred in the cycle. Therefore, it is necessary to study the fundamental reasons behind the aneuploidy to explore new strategies for improvement.

In previous studies, several causes of aneuploidy have been proposed, including nondisjunction of homologous chromosomes^13^, premature separation of sister chromatids during the first meiotic division^14^, anaphase lag^15^ and congression failure^15^. Cytokinesis is the last step of cell division that physically separates the daughter cells. Cytokinesis failure has also been implicated as a contributor to aneuploidy. Sgura et al. observed centromere-positive micronuclei and chromosome nondisjunction as indicators of aneuploidy in lymphocytes after cytokinesis was blocked^16^. Kamino et al. found increased cytokinesis and aneuploidy in cells following nuclear DNA damage^17^, and Rosario et al. obtained similar results^18^. Salem et al. showed that the mouse embryonic fibroblast exhibited polyploidy and failure to undergo cytokinesis when the Nek7 gene was knocked out^19^. Gisselsson et al. found that cancer cells exhibited trisomy due to multipolar mitosis and incomplete cytokinesis^20^. Högnäs et al. suggested that cytokinesis failure induces aneuploidy and conversion of non-transformed cells to tumourigenic cells *in vitro* and *in vivo*^21^. A tight interaction was indicated between aberrant cytokinesis, mitosis failure and apoptosis^22^. However, to our knowledge, few studies have investigated the effects and molecular mechanism of cytokinesis failure on aneuploidy in human oocytes and embryos at a single-cell level.

In the present study, we investigated important genes showing dynamic expression changes during the fertilisation process using single-cell sequencing data. Cytokinesis-related genes were screened and identified via single-cell real-time PCR methods. The expression levels of the screened genes were identified in oocytes and in the resulting embryos from *in vivo*-matured (IVO) and IVM oocytes, and the functions of these genes were analysed via siRNA knock-down.

## Results

### Screening of important genes before and after fertilisation in oocytes

**Figure 1:**
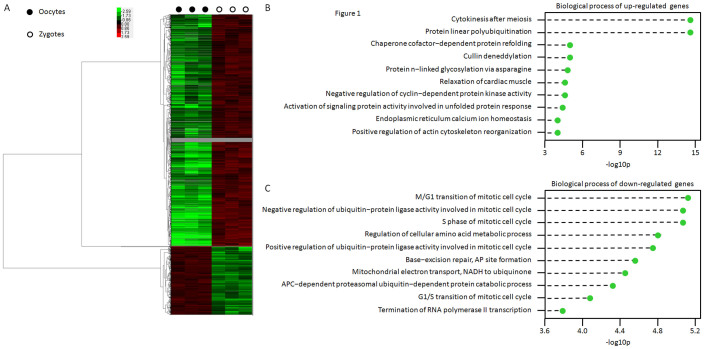
Analysis of differentially expressed genes during fertilisation. (A) Supervised clustering analysis based on the differentially expressed genes between oocytes and zygotes; (B) Top 10 biological processes of up- and down-regulated genes.

In total, 2233 genes were shown to be significantly up- or down-regulated between oocytes and zygotes. Of those, 1717 genes were up-regulated, and 516 genes were down-regulated, and the cluster analysis showed an obvious distinction between oocytes and zygotes (Figure 1A) (Supplementary data 2). Moreover, Gene ontology (GO) analysis indicated that the up-regulated genes were involved in 171 different biological functions and 78 molecular functions, and the down-regulated genes were involved in 124 biological functions and 25 molecular functions (Supplementary data 3). The top 10 biological functions of the up-regulated and down-regulated genes are shown in Figure 1 B and C, respectively. Of the changed biological processes, the top dynamic changes were related to cell cycle control, which indicates the importance of the transition of the cell cycle from meiosis to mitosis in the zygote stage. Furthermore, 31 pathways showed dynamic changes that were impaired by up-regulated genes, and 20 pathways displayed dynamic changes that were impaired by down-regulated genes. The top 10 pathways impaired by differentially expressed genes are shown in Figure S1.

### Identification of cytokinesis-related genes in normal human oocytes and zygotes

**Figure 2:**
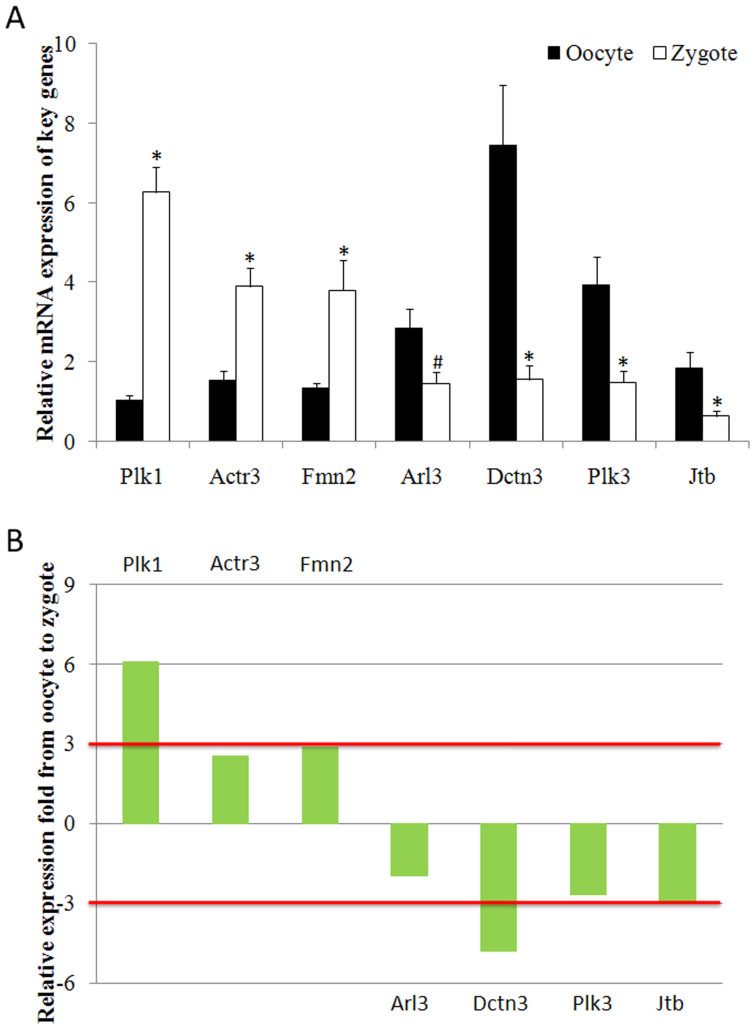
Expression of cytokinesis-related genes in oocytes and zygotes. (A) Expression levels of Actr3, Plk1 and Fmn2 were significantly increased, but those of Arl3, Dctn3, Plk3 and Jtb were significantly decreased after fertilisation. * indicates significant differences between oocyte and zygote and a ratio reaching 2-fold (P<0.05), and ^#^ indicates significant differences between oocyte and zygote (P<0.05), but the ratio did not reach 2-fold; (B) Only 3 genes showed more than 3-fold dynamic changes during fertilisation: Plk1, Dctn3 and Jtb.

Cytokinesis is a key biological process in zygotes and is closely associated with cell division and chromosome separation. A total of 7 genes were involved in the process of cytokinesis during fertilisation, including 3 up-regulated genes (Actr3, Plk1 and Fmn2) and 4 down-regulated genes (Arl3, Dctn3, Plk3 and Jtb). Thirty matured human oocytes and 37 human zygotes were collected, and the expression levels of the 7 genes were identified in these samples. The results indicated that Plk1, Actr3 and Fmn2 gene expression levels were significantly increased by 6.09-fold, 2.54-fold and 2.86-fold, respectively, in zygotes compared with oocytes. With regard to the down-regulated genes, expression level changes of 1.95-fold for Arl3, 2.68-fold for Plk3, 4.79-fold for Dctn3, and 3.01-fold for Jtb were observed in oocytes compared with zygotes (Figure 2). Therefore, because of their significant dynamic expression changes during fertilisation, Plk1, Jtb and Dctn3 were selected for further study.

### Identification of cytokinesis-related genes in human zygotes arrested at the pronuclear and 8-cell stages

**Figure 3:**
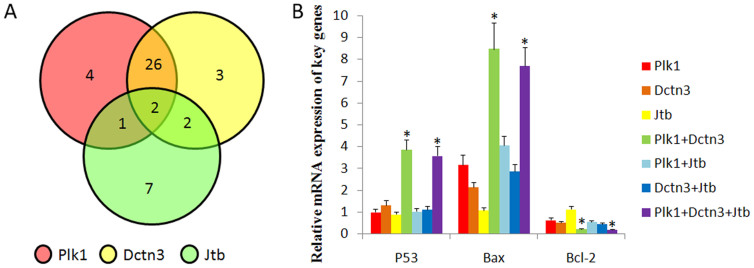
Screening of key genes in human zygotes arrested at the pronuclear stage. (A) The number of embryos that expressed Plk1, Dctn3, Jtb, Plk1 and Dctn3, Plk1 and Jtb, Dctn3 and Jtb, and all three genes. (B) Expression of apoptosis-related genes in arrested zygotes with different gene expression levels of Plk1, Dctn3, Jtb and their combinations. A total of 7 groups were identified according to the types and number of differentially expressed genes. Only 2 of these groups show significant differential expression of apoptosis genes in the corresponding embryos, the “Plk1+Dctn3” and “Plk1+Dctn3+Jtb” groups. * indicates significant differences between oocytes and zygotes with a ratio reaching 2-fold (P<0.05).

To ensure that the genes play important roles in the first cleavage event in human embryos, the expression of Plk1, Jtb and Dctn3 in a total of 48 zygotes arrested at the pronuclear stage was tested. The results suggested that 45 of the 48 arrested zygotes displayed aberrant gene expression of at least one of the screened genes. Notably, 26 arrested zygotes showed abnormal expression levels of Plk1 and Dctn3 to a significantly higher extent than did the other groups (Figure 3A). Moreover, we found that expression of apoptosis-related genes, including P53, Bax and Bcl2, were dramatically changed in the zygotes with abnormal expression of Plk1 and Dctn3, regardless of the Jtb expression levels (Figure 3B).

**Figure 4:**
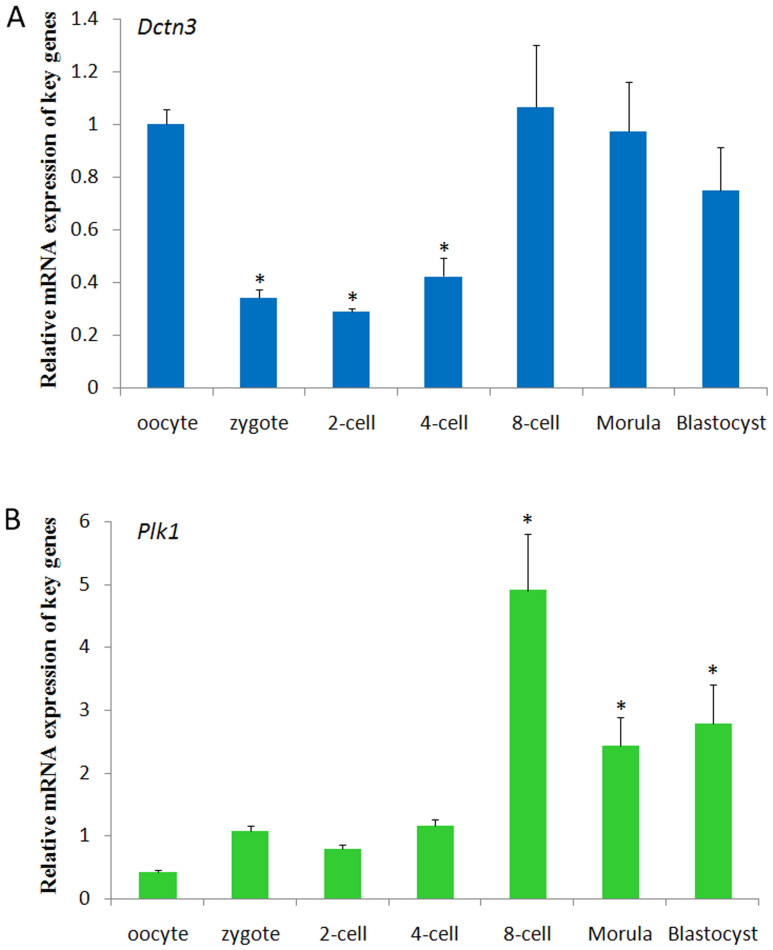
Expression of Plk1 and Dctn3 in the early developmental stages (human oocytes and resulting embryos). (A) Dctn3 expression characteristics were shown, and higher expression levels were observed in oocytes, 8-cell, morula and blastocyst stages. * indicates significant differences between oocytes and zygotes (P<0.05); (B) Plk1 expression characteristics were shown, and higher expression levels were observed in 8-cell, morula and blastocyst stages. * indicates significant differences between oocytes and zygotes (P<0.05).

To assess the role of cell division in embryonic development, we identified changes in gene expression between oocytes and blastocysts. The results indicated that Dctn3 showed higher expression in oocytes but lower expression in the subsequent three stages, the zygote, 2-cell and 4-cell stages. The expression levels then increased again from the 8-cell stage to the blastocyst stage (Figure 4A). For Plk1, the expression pattern was similar to Dctn3 except in oocytes; lower expression of this gene was observed in MII oocytes (Figure 4B).

To analyse Plk1 and Dctn3 expression at the 8-cell stage, a key stage for human fertilised embryos, 38 human embryos arrested at the 8-cell stage were collected; half of each embryo was used to identify gene expression levels, and the other half was used to identify the chromosome karyotype. The results indicated that 27 arrested embryos had aberrant expression levels of both Plk1 and Dctn3 and that the remaining 11 arrested embryos had either abnormal Plk1 (4 embryos) or abnormal Dctn3 (7 embryos) expression levels (Figure S2 A). Twenty-five embryos with aberrant Plk1 and Dctn3 gene expression showed a chromosome microdeletion/duplication, and 9 of the other 11 embryos did as well (Figure S2 B).

To further examine the role of Plk1 and Dctn3 expression in embryonic development, we also determined Plk1 and Dctn3 expression levels in blastocysts of high or poor quality. A total of 22 high-quality and 32 poor-quality blastocysts were collected. Sixteen of the 22 high-quality blastocysts showed normal Plk1 and Dctn3 gene expression levels, 4 of them showed aberrant expression of either Plk1 or Dctn3, and 2 of them showed aberrant expression of both Plk1 and Dctn3. However, the proportion of embryos with normal Plk1 and Dctn3 gene expression in poor-quality blastocysts was significantly decreased compared with high-quality blastocysts (5/32 poor-quality vs. 16/22 high-quality), and the number of embryos with abnormal Plk1 and/or Dctn3 gene expression in poor-quality blastocysts was significantly increased (27/32 poor-quality vs. 6/22 low-quality) (Figure S3 A). Moreover, of the 33 blastocysts with aberrant Plk1 and/or Dctn3 gene expression, 27 showed a chromosome microdeletion/duplication, while of the 21 blastocysts with normal Plk1 and Dctn3 gene expression, only 4 showed a chromosome microdeletion/duplication (Figure S3 B).

### Aberrant co-expression of Plk1 and Dctn3 in human IVM oocytes contributes to aneuploidy and developmental failure in fertilised embryos

**Figure 5:**
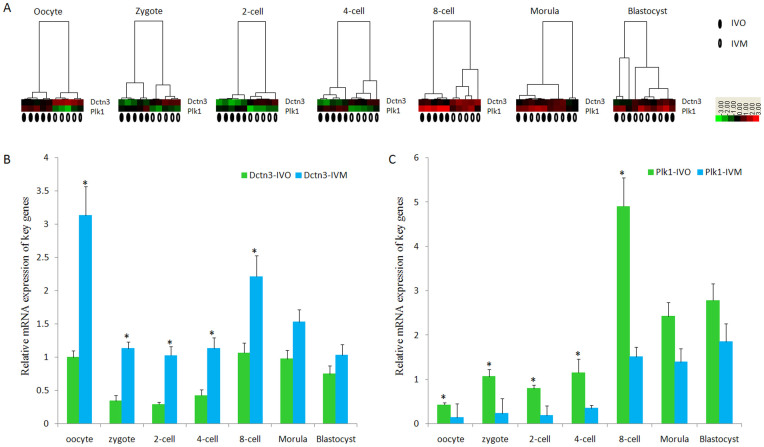
Comparison of expression level of Plk1 and Dctn3 between *in vivo* matured (IVO) and *in vitro* matured (IVM) oocytes. (A) Cluster analysis of both genes in different developmental stages, including oocyte, zygote, 2-cell, 4-cell, 8-cell, morula and blastocyst stages. Before the morula stage, distinct differences of expression levels of both genes were observed in IVO and IVM oocytes, but there was no difference between these two samples in the morula and blastocyst stages. (B) Dctn3 expression levels in IVM oocytes were significantly increased in the oocyte, zygote, 2-cell, 4-cell and 8-cell stages, but no differences were found in the morula and blastocyst stages compared with those of IVO oocytes. * indicates significant differences between oocytes and zygotes with a ratio reaching 2-fold (P<0.05); (C) Plk1 expression levels in IVM oocytes were significantly decreased in the oocyte, zygote, 2-cell, 4-cell and 8-cell stages, but no differences were found in the morula and blastocyst stages compared with those of IVO oocytes. * indicates significant differences between oocytes and zygotes with a ratio reaching 2-fold (P<0.05).

Embryo aneuploidy and developmental arrest at zygotic genome activation (ZGA) (8-cell stage)^23^ has often been observed in human embryos from IVM oocytes^24^. Therefore, expression profiling of both Plk1 and Dctn3 was carried out in IVM oocytes and the resulting embryos. The cluster data from the gene expression analysis suggested that distinct differences for both genes were found between the samples from IVO and IVM oocytes before the morula stage. However, the expression profiles were mixed and indistinct at the morula and blastocysts stages (Figure 5A). The PCR results suggested that gene expression levels of Dctn3 were increased in IVM oocytes and embryos at the zygote, 2-cell, 4-cell and 8-cell stages, but not in the morula and blastocyst stages. Further, Plk1 expression was decreased in IVM oocytes and embryos at the zygote, 2-cell, 4-cell and 8-cell stages, but not in the morula and blastocyst stages, compared with IVO oocytes and the corresponding resulting embryos (Figure 5B and C). To determine whether the differences were specifically attributed to the IVM procedure, we enlarged the sample size for IVO and IVM oocytes, and the results suggested significant differences between the groups (Figure S4).

**Figure 6:**
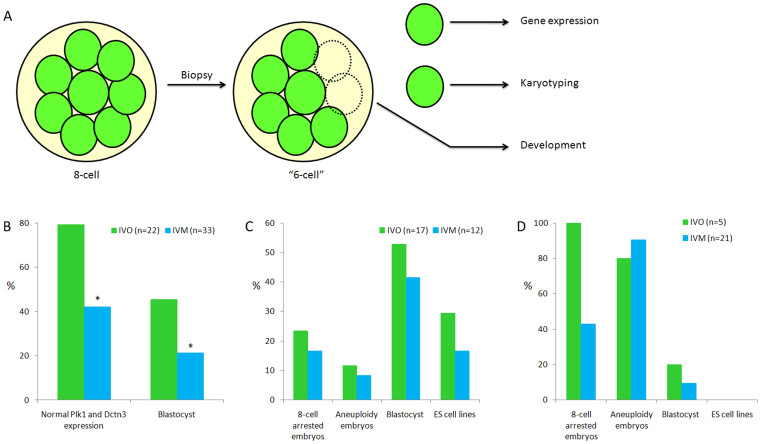
Identification of gene expression levels, embryo aneuploidy and developmental potential in single 8-cell embryos. (A) Schematic figure for the testing procedure; (B) The accuracy of Plk1 and Dctn3 expression levels as well as blastocyst formation rates were all significantly higher in the embryos from IVO oocytes compared with those from IVM oocytes. * indicates significant differences between oocytes and zygotes (P<0.05); (C) No differences were observed between the embryos with normal Plk1 and Dctn3 expression from IVO or IVM oocytes, including arrested embryos at the 8-cell stage and aneuploid embryos, as well as in blastocyst formation and embryonic stem cell derivation; (D) No differences were shown between the embryos with abnormal Plk1 and Dctn3 expression from IVO or IVM oocytes, and no embryonic stem cell lines could be derived from such blastocysts.

Considering the importance of the 8-cell stage as the stage at which embryos are transferred in the clinic and at which zygotic genome activation ends, twenty-two and 33 human 8-cell stage embryos developed from IVO and IVM oocytes, respectively, were collected for further study. One blastomere was collected from each 8-cell embryo to perform gene expression analysis, and another blastomere was used for CGH testing. The residual “6-cell” embryos were cultured to the blastocyst stage (Figure 6A). Twenty-two and 33 biopsied embryos were obtained from IVO and IVM oocytes, respectively. Among these embryos, almost 80% of the embryos from IVO oocytes showed normal Plk1 and Dctn3 expression levels, which was a significantly higher proportion than that observed in IVM oocytes. The blastocyst developmental efficiency also displayed this tendency (Figure 6B). Moreover, in those embryos with normal Plk1 and Dctn3 expression, there were no significant differences among the proportions of embryos arrested at the 8-cell stage, embryos with aneuploidy and blastocysts formed (Figure 6C). Further, in those embryos with abnormal Plk1 and Dctn3 expression levels, high proportions of embryos arrested at the 8-cell stage and of aneuploid embryos (based on karyotyping) were observed. No embryonic stem cell lines were derived from the blastocysts of such embryos (Figure 6D).

### Dctn3 regulated Plk1 expression to maintain embryonic euploidy

**Figure 7:**
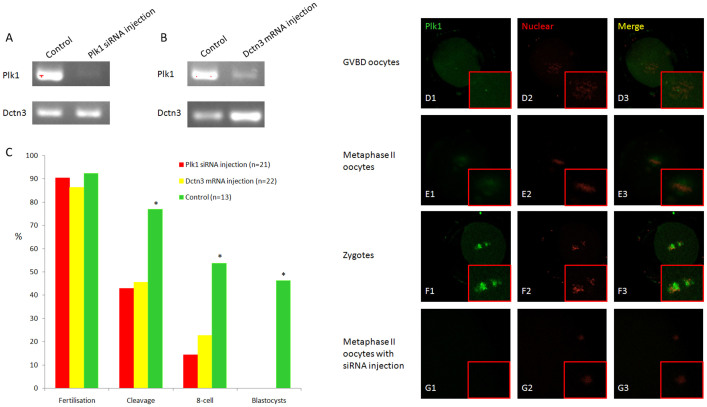
Functional analyses of Plk1 and Dctn3 genes in IVO oocytes. (A) Plk1 siRNA induced the silence of Plk1 gene but not Dctn3 gene expression; (B) *In vitro*-transcribed mRNA injection induced overexpression of Dctn3 and also induced silencing of Plk1; (C) Fertilisation rate was not impaired by Plk1 siRNA injection or Dctn3 mRNA injection, but the efficiency of cleavage, 8-cell and blastocyst formation were decreased in both treatment groups. * indicates significant differences between oocytes and zygotes (P<0.05). PLK1 expression was identified by immunofluorescence; (D) PLK1 began to aggregate around the condensed chromatin after GVBD; (E) PLK1 localised to the two poles in metaphase II oocytes; (F) After fertilisation, PLK1 accumulated between the two pronuclei and began to be separated during pronuclear breakdown; (G) Plk1 siRNA injection inhibited PLK1 protein expression.

To study the function of Dctn3 and Plk1 in human embryos and corroborate above results, we injected Plk1 siRNA and Dctn3 mRNA into human *in vivo*-matured oocytes. The PCR results from oocytes injected with Plk1 siRNA indicated that Plk1 mRNA was weakly expressed and difficult to detect. However, Dctn3 expression was not impaired (Figure 7A). *In vitro*-transcribed Dctn3 mRNA was injected into 22 oocytes. We found that Dctn3 expression was up-regulated but Plk1 expression was down-regulated (Figure 7B). We found that these oocytes can be fertilised with normal efficiency, but the cleavage efficiency decreased from approximately 78% in the control group to 43–45% in the Plk1 or Dctn3 RNAi groups. Development of all of the embryos arrested at the 8-cell stage, and all embryos showed abnormal karyotypes (Figure 7C). Representative images for PLK1 location at GVBD, MII and zygote stages are shown in Figure 7D–F. After Plk1 siRNA injection, much lower Plk1 signal was detected in the IVO MII oocytes (Figure 7G). The representative images for Plk1-siRNA injected oocytes were shown in Figure S5 A, which characteristics looks similar with IVM embryos with lower quality (Figure S5 B), but very different from IVM embryos with higher quality (Figure S5 C).

## Discussion

The present study investigated the dynamic changes in gene expression that occur during fertilisation, and key genes and pathways, including the cytokinesis pathway, were suggested to play a role. Two genes, Plk1 and Dctn3, were identified successfully using human oocytes, and they both seemed to be important for embryo cleavage and maintenance of chromosome euploidy. Furthermore, normal expression of both genes was correlated with embryo chromosome aneuploidy and developmental potential, suggesting an important role for both genes in the developmental regulation of human IVM oocytes. The function of these two genes was studied by siRNA injection, and the results facilitate a better understanding of human oocyte fertilisation and embryonic development that can be used to improve developmental efficiency of human IVM oocytes.

Omics technology has been applied to screen significant molecular markers from biological samples, and at present, genomics, transcriptomics, proteomics and metabolomics have been successfully applied in the fields of biology and medicine^25^. Transcriptomics for mRNA analysis can help to identify valuable genes in the early stages of development. The high-throughput data from our previous study were compared with gene expression changes from the oocyte to blastocyst stages using single-cell transcriptome sequencing technology^26^. Single-cell RNA amplification and identification techniques were developed in 2009^27^ and have gradually become popular in studies on embryonic development^26^,^28^ and stem cell biology^29^. There are some advantages of single-cell RNA testing, e.g., gene expression can be identified using only a small RNA volume and individual differences can be compared. In the present study, we identified gene expression levels using single oocytes, zygotes and even blastomeres using this technique, which was more sensitive and accurate than immunofluorescence.

Traditional methods of studying molecular mechanisms during these early developmental stages normally depend on immunofluorescence staining or real-time PCR using pooled oocytes. Our lab has established a single-cell sequencing platform to analyse RNA expression patterns in oocytes and embryos^26^. From the published data, we identified genes whose expression profiles in oocytes differed from those in zygotes; such genes may play a key role during fertilisation. Of more than 2,200 differentiated genes, approximately 300 genes were involved in the cell cycle and cell division. These results describe for the first time changes to molecular dynamics in the whole transcriptome during the transition from oocytes to zygotes.

In mice, the first embryonic cell cycle relies on maternal factors, including mRNA and proteins. However, in humans, the first three cell cycles depend on maternal factors because zygotic genomic activation is not completed until the 8-cell stage^30^. Therefore, maternal cytokinesis-related molecular markers appear to be more important in supporting the transition from meiosis to mitosis and maintaining the normal mitosis process at early developmental stages. In the present study, a total of 7 maternal cytokinesis-related genes were screened by bioinformatics, and three of them were identified to have similar expression states as in the sequencing results: Plk1, Dctn3 and Jtb. Of these three genes, both Plk1 and Dctn3 were suggested to play roles in cell cycle regulation but had not yet been studied in human fertilised embryos.

The Dctn3 gene and its function were described in 1998^30^. Dctn3 was suggested to play roles in ER-to-Golgi transport, the centripetal movement of lysosomes and endosomes, spindle formation, cytokinesis, chromosome movement, nuclear positioning, and axonogenesis. Dctn3 encodes one subunit of dynactin, which consists of 10 subunits in total, and is responsible for the attachment of DCTN1 to DCTN2^31^. In humans, Dctn3 is located on chromosome 9, consists of 7 exons^32^, and has similar functions as in mice where it, together with dynein, is involved in regulating mitosis. However, many studies on Dctn3 in cell division were performed in somatic cells, embryonic stem cells, and even tumour cells^33^,^34^,^35^ while few studies have focused on embryonic mitosis, especially in the context of human embryonic development. Payne et al. suggested that the DCTN1 subunit is important in perinuclear-mediated genomic union during mammalian fertilisation through an association with nuclear pore complex proteins^36^. Regarding the key role of DCTN3 in combining with DCTN1 and the other subunits, DCTN3 may be responsible for accurate spindle assembly. Our results indicate that Dctn3 is overexpressed in arrested zygotes. A previous study suggested that the dynactin complex is disrupted following overexpression of one of its subunits^37^, leading to impaired spindle assembly and microtubule aggregation. Recently, the dynactin complex was suggested to play a new role in regulating the spindle assembly checkpoint by recruiting PLK1 to kinetochores and facilitating phosphorylation of important downstream targets^38^. In the present study, Plk1 and Dctn3 were also found to be aberrantly expressed in arrested zygotes, suggesting that they have important roles in early embryos.

Plk1 is an early trigger for the G2/M transition. During interphase, PLK1 localises to centrosomes. In early mitosis, it associates with the mitotic spindle poles and has been suggested to localise to the centromere/kinetochore region, suggesting a possible role for chromosome separation^39^. In humans, PLK1 deletion by antibody injection resulted in the failure of HeLa cell division^40^, consistent with our results. When we injected siRNA into oocytes, the injected oocytes were often unable to finish dividing after fertilisation. Further, lower Plk1 expression levels were also identified in arrested zygotes. A possible mechanism for these observations is that DCTN3 is essential to bridge DCTN1 and DCTN2^41^, therefore increasing expression of DCTN3, which may result in an increased abundance of dynactin complex containing DCTN2. However, DCTN3 binds to endogenous competitive subunits when DCTN2 expression is upregulated. Such binding destabilises the shoulder structure that links the DCTN1 arm to the ARP1 filament and leads to complex disturbance^42^, which thus reduces the expression of DCTN6. The decreased DCTN6 levels would then lead to a reduction in PLK1 recruitment and thus a decrease in Plk1 expression.

Plk1 is considered a proto-oncogene; overexpression of Plk1 is often observed in tumour cells, and Plk1 acts as a target for cancer drugs^43^. In our study, higher expression of Plk1 was suggested in the embryos with abnormal division speed, such as 4-cell embryos at day 2, or 12-cell embryos at day 3 (data not shown). Regardless of whether Plk1 was over- or underexpressed, aberrant expression of Plk1 in embryos resulted in lower developmental potential.

Developmental failure can result from aneuploidy, which is often induced by aberrant centrosome or centromere/kinetochore constitution. Plk1 regulates centrosome duplication and maturation from late S phase to prophase, and abnormal Plk1 expression can lead to impaired centrosome size or number. In some tumour cells, abnormal centrosome amplification induced by over-expressed Plk1 leads to multipolar spindles and results in unequal segregation of chromosomes^44^. Furthermore, Plk1 expression was observed in the centromere/kinetochore region in early mitosis and was shown to be regulated by the dynactin complex, indicating a potential important role in centromere/kinetochore configuration^38^,^45^. Therefore, reduced expression of Plk1 would result in the loss of centromeres and a consequent random distribution of chromosomes into daughter cells, yielding aneuploid karyotypes. Aneuploidy seems to be a normal phenomenon in developing embryos^15^, which suggests early developmental inefficiency in humans, especially for human IVM oocytes^8^. In our study, we demonstrated that 8-cell embryos from IVM oocytes seem to have an equivalent developmental potential as those from IVO oocytes as long as they have normal expression levels of Plk1 and Dctn3. Considering the dynamic changes of Plk1 and Dctn3 in IVM oocytes and the subsequent development stages until the 8-cell stage, differences in maternal gene expression contribute to the lower developmental potential of IVM oocytes. Spindle assembly mistakes have been explored in a previous study^46^, and some important molecular markers of cell cycle checkpoints have been implicated^47^,^48^. However, our study is still the first to examine Plk1 and Dctn3 in human oocytes and show that the co-expression of both genes is key to IVM developmental potential.

As a potential technique, IVM has been applied in clinical settings to help patients who are susceptible to OHSS or who suffer from ovarian dysfunction; however, this technique has not been widely applied because of its poor outcome compared with COH cycles. One possible explanation for its poor outcome is that the endocrinological, endometrial, and other conditions in IVM patients are weaker than in patients with tubal factors or for whom infertility can be attributed to male factors. Another possible explanation is that *in vitro* environments do not accurately mimic *in vivo* conditions and therefore cause a decline in the developmental competence of IVM oocytes. An *in vitro* maturation environment would impair not only spindle assembly and cytoplasm maturation but also DNA epigenetic modifications and subsequent RNA transcription and protein expression^49^,^50^. To improve outcome, some have modified the IVM process, for example, by supplementing with biomolecules^51^, or hormones^52^ or by adjusting maturation duration^53^. However, observed improvements still lack confirmation at a molecular level and identification of a molecular target. Our present study suggests that both Plk1 and Dctn3 have key roles in regulating embryo quality, especial for IVM embryos. Plk1 and Dctn3 have potential as molecular targets in future efforts to improve the developmental competence of IVM oocytes and resulting embryos.

Aside from Plk1 and Dctn3, some other genes have also been suggested to have key roles in and to exhibit dynamic changes during oocyte maturation in an *in vitro* environment^54^. Such dynamic changes in gene expression indicate the effects of IVM culture on RNA transcription and protein translation. These studies were primarily performed in rodents and domestic animals; however, expression of these genes is likely also altered in human IVM oocytes because of the high degree of similarity in the IVM culture environment and media used for embryos of different species. Epigenetic modifications were found to be responsible for these changes in recent studies, and changes in methylation of imprinted genes would impair the development of IVM oocytes^50^. Therefore, it is better to seek more key molecules, confirm their function and understand their mechanisms of regulation, which will help improve the development of IVM oocytes.

## Conclusion

In the present study, we screened important cytokinesis-related molecular markers during fertilisation and subsequent developmental processes and determined their expression in normal and abnormal human embryos at different stages. Importantly, abnormal expression of Plk1 and Dctn3 in 8-cell embryos was closely correlated with aneuploidy, which significantly reduces the developmental potential of embryos. This correlation was more apparent in embryos from human IVM oocytes. Therefore, the co-expression of Plk1 and Dctn3 may be useful as a marker of good quality embryos with normal karyotypes during IVM therapy using the PGS method and in further IVM studies. The use of Plk1 and Dctn3 expression as a marker may eventually facilitate improved outcomes for assisted reproductive technology.

## Methods

The human tissue collection and study procedure were approved by the Institutional Review Board at Peking University Third Hospital in the present study, and the methods used in the present study followed closely the guidelines legislated and posted by the Ministry of Health of the People's Republic of China. The patients involved in the present study were informed of all details of the procedure, including sample utility and research destination, and voluntarily signed an informed consent document.

### Screening of differentially expressed genes for single-cell mRNA transcriptome raw data

Raw data from our previously published study were used^26^. Genes that were differentially expressed between oocytes and zygotes were analysed using DEGseq using the R statistics software as described in a previous study^55^; a threshold of FC (Fold Change) ≥2 or ≤0.5 was adopted.

### Unsupervised and supervised hierarchical clustering

Heatmaps and hierarchical clustering were performed using TM4 MeV from TIGR or the Partek Genomic Suite using z-scores transformed from the original normalised values. Following the unsupervised hierarchical clustering method, Plk1 and Dctn3 were analysed using supervised clustering.

### Gene ontology (GO) and pathway analysis for differentially expressed genes

For GO analysis, the p value of each GO term was calculated using the hyper-geometric distribution (HGD) method and was corrected by multiple testing methods to control for false discovery rate (FDR). Then, redundant GO terms were removed from the total GO terms in the threshold range, and the terminal GO terms in the hierarchy chart were selected.

For pathway analysis, the Kyoto Encyclopedia of Genes and Genomes (KEGG) database was used. A calculation method similar to the GO analysis was performed, and selected genes (FDR [5%]) were then analysed using the KEGG database to identify specific biological pathways.

### Collection of human oocytes matured *in vivo* and *in vitro*

For the collection of human IVO oocytes in the present study, four women who had conceived at least one healthy baby voluntarily donated their oocytes. No financial benefit was involved in the donation process. The egg donors were informed of all details of the procedure, including egg utility and research destination, and all voluntarily signed a series of informed consent documents. All of the eggs were used in basic scientific research. All of the procedures closely followed the guidelines legislated and posted by the Ministry of Health of the People's Republic of China. The IVO oocytes were collected after gonadotropin stimulation and cultured at 37.0°C and 5% CO_2_ in humidified air.

For human IVM oocytes, patients undergoing intra-cytoplasmic sperm injection (ICSI) treatment at the Peking University The Third Hospital between January 2013 and December 2013 were included in this study. Oocytes that had failed to mature following controlled ovarian hyperstimulation and were either at the germinal vesicle (GV) stage (defined by the presence of a GV structure) or MI stage (defined by the absence of both a polar body and a GV structure) were collected and cultured *in vitro*. Immature oocytes from different patients were randomly allocated to the various experimental groups. Immature oocytes were cultured in commercial IVM medium supplemented with 75 mIU/ml FSH, LH and EGF-BDNG-IGF1 growth factors, as described in our previous study^56^. After 24 or 36 hours, oocytes that had expelled the first polar body were collected and prepared for further experiments.

### Immunofluorescence

The location of these differentially expressed genes was identified by immunofluorescence. The oocytes or zygotes were first rinsed and fixed in 4% paraformaldehyde for 20 min, permeablised with 0.2% Triton X-100 for 30 min and blocked in 3% BSA in PBS for 2 h at room temperature. Incubation with primary antibody was carried out overnight at 4°C. After rinsing, the embryos were incubated under the same conditions with fluorescein isothiocyanate (FITC)-conjugated secondary antibody for 2 h. The nuclear status of the embryos was evaluated by staining with 10 μg/mL propidium iodide for 10 min. Finally, the embryos were mounted on glass slides and examined with a confocal laser scanning microscope (LSM710, Zeiss, Germany). Anti-PLK1 antibodies (37-7000, Invitrogen Corp., USA) were used at a dilution of 1:100.

### Gene function analysis by gene knockdown and overexpression

siRNA was used to inhibit gene expression. The siRNA powder was diluted using DEPC water and then injected into the MII oocytes. Successful inhibition was confirmed by RT-PCR. For overexpression, the mRNA was restored in an alcohol solution, which was removed by centrifugation prior to injection. The *in vitro* mRNA transcripts were injected into the MII oocytes following a similar procedure as that performed for siRNA injection. Oocytes injected with DEPC water were used as controls.

### Aneuploidy analysis by the CGH method

Genomic DNA from blastomeres was amplified using a whole-genome amplification kit. CGH manipulation using the BlueGnome platform was performed according to the instruction book and as described previously^57^. The amplified DNA samples were labeled with Cy3 or Cy5 using a fluorescent labelling system and mixed with control human DNA. The samples were then dried, resuspended and loaded onto BlueGnome 24Sure V3 arrays. The data were analysed using Bluefuse software (BlueGnome, Illumina Inc., USA).

### Gene expression identification by RT-PCR

RNA from single cells was prepared using a commercial kit (SMARTer® Ultra Low Input RNA for Illumina® Sequencing, Clontech® Laboratories, Inc., USA). Total RNA was extracted, and the first-strand cDNA was synthesised. Quantitative real-time RT-PCR was then performed using an ABI 7500 machine (Applied Biosystems, USA). Sample cells from three plates were run in duplicate, using the threshold suggested by the software for the instrument to calculate Ct. To normalise readings, we used Ct values from Gapdh as internal controls for each run, obtaining a delta Ct value for each gene. Relative changes in gene expression data were analysed by the 2^−ΔΔCT^ method. Primer sequences are shown in Supplementary Table 1.

We calculated the averaged expression levels of Plk1 and Dctn3 for all oocytes in the IVO group. If the expression level for each gene was in a range from 0.5- to 2-fold of the average expression level in a single cell, the embryo was regarded as having normal Plk1 and Dctn3 expression. If the expression level in any one of the genes was insufficient or excessive compared with average level in a single cell, the embryo was considered abnormal.

### Statistical analysis

The results were compared using SPSS software (Chicago, IL, USA). An independent sample T test was performed when comparing the gene expression data from two groups. A one-way ANOVA test was used when comparing the data from more than three groups. A χ^2^ method was applied when comparing the number of embryos between two groups. P values of < 0.05 were considered to indicate significant differences.

## Author Contributions

Y.F., C.H.Z. and Q.J.L. performed the experiments, and was also took part in manuscript drafting, critical discussion and data analysis. T.T. performed bioinformatics analysis. T.D. performed micromanipulation experiment and R.L. charged for human oocytes collection. Y.Z., J.Y. and F.X.S. took part in critical discussion and data analysis. Y.Y. joined in study design, manuscript drafting and critical discussion and manuscript submission. J.Q. contributed to the conception of design, coordinated the research and manuscript editing.

## Supplementary Material

Supplementary Information supplementary metarials

Supplementary Information Dataset 1

Supplementary Information Dataset 2
