# Supplementary material for: Aberrant expression of maternal Plk1 and Dctn3 results in the developmental failure of human in-vivo- and in-vitro-matured oocytes
Source: Sci Rep. 2015 Feb 3;5:8192. doi: 10.1038/srep08192 (PMC4314639; doi:10.1038/srep08192)
Supplement: Supplementary Information — supplementary metarials [file srep08192-s1.pdf]

Aberrant expression of maternal Plk1 and Dctn3 results in the developmental failure of human *in-vivo*- and *in-vitro*-matured oocytes

Yong Fan[1#], Hong-Cui Zhao[2, 3, 4#], Jianqiao Liu[1#], Tao Tan[5], Ting Ding[2, 3], Rong Li[2, 3, 4], Yue Zhao[2, 4], Jie Yan[2, 3], Xiaofang Sun[1], Yang Yu[2, 3, 4 *], Jie Qiao[2, 3, 4 *]

1 Key Laboratory for Major Obstetric Diseases of Guangdong Province, The Third Affiliated Hospital of Guangzhou Medical University, Guangzhou, 510150, China

2 Center of Reproductive Medicine, Department of Obstetrics and Gynecology, Peking University Third Hospital, Beijing, 100191, China

3 Key Laboratory of Assisted Reproduction, Ministry of Education, Beijing, 100191, China

4 Beijing Key Laboratory of Reproductive Endocrinology and Assisted Reproductive Technology, Beijing, 100191, China

5 Yunnan Key Laboratory of Primate Biomedical Research and Kunming Biomed International and National Engineering Research Center of Biomedicine and Animal Science, Kunming, 650500, China.

Supplementary figure legends

Figure S1 Top 10 pathway processes of up- and down-regulated genes.

Figure S2 Distribution of expression of Plk1 and Dctn3 in human arrested 8-cell embryos and the resulting aneuploidy. (A) In arrested 8-cell embryos, approximately 71% of embryos showed aberrant co-expression of Plk1 and Dctn3, 11% of embryos showed only the aberrant expression of Plk1, and 18% of embryos showed only the aberrant expression of Dctn3; (B) Higher aneuploidy was found in the arrested embryos with abnormal expression of Plk1 or/and Dctn3.

Figure S3 Effects of Plk1 or/and Dctn3 expression levels on embryo quality and chromosome aneuploidy. (A) Nearly 80% embryos have the higher quality in Plk1+Dctn3 normal expression group, but only 10% embryos have the similar quality in Plk1+Dctn3 abnormal. When only Plk1 or Dctn3 expression was abnormal, about half of embryos were graded as higher quality. (B) Abnormal expression of Plk1 or/and Dctn3 also significantly impaired chromosome ploidy in embryos. The number in the column means the quantity of embryos in each group.

Figure S4 Relative mRNA expression of Plk1 and Dctn3 in *in-vitro* and *in-vivo* matured oocytes. (A) Dctn3 expression level was significantly increased, and (B) Plk1 expression level was significantly declined in IVM oocytes. * indicates significant differences between *in-vitro* and *in-vivo* matured oocytes.

Figure S5 Representative images for embryo morphologies at different developmental stages with or without Plk1-siRNA injection. In the embryos with Plk1-siRNA injection, the morphologies at zygote (A), 2-cell (A1), 4-cell (A2) and 8-cell (A3) stage looks similar with the lower quality IVM embryos without Plk1-siRNA injection (B-B3). No blastocysts were derived from both groups (A4 and B4). Some fertilized embryos without Plk1-injection displayed the normal morphologies at zygote (C), 2-cell (C1), 4-cell (C2) and 8-cell (C3) stage, and high quality blastocyst can be obtained from them (C4).

Table S1 Primer sets used for real-time PCR

Gene	Primer Sets(5' to 3')
P53	Forward: 5′-GGAAATTTGTATCCCGAGTATCTG-3′
	Reverse: 5′-GTCTTCCAGTGTGATGATGGTAA-3′
Bax	Forward: 5′-AGTAACATGGAGCTGCAGAGG-3′
	Reverse: 5′-ATGGTTCTGATCAGTTCCGG-3′
Bcl2	Forward: 5′-GTGACTTCCGATCAGGAAGG-3′
	Reverse: 5′-CTTCCAGACATTCGGAGACC-3′
Oct4	Forward: 5′- CTTGCTGCAGAAGTGGGTGGAGGAA-3′
	Reverse: 5′- CTGCAGTGTGGGTTTCGGGCA-3′
Nanog	Forward: 5′- CAGCTGTGTGTACTCAATGATAGATTT -3′
	Reverse: 5′- CAACTGGCCGAAGAATAGCAATGGTGT -3′
Rex1	Forward: 5′- GCTGACCACCAGCACACTAGGC-3′
	Reverse: 5′- TTTCTGGTGTCTTTGCCCG-3′
Actr3	Forward: 5′- CAATCCTTGGAAACTGCTA-3′
	Reverse: 5′- CCATTTTGACCCATCTGTA-3′
Plk1	Forward: 5′- TTCGTGTTCGTGGTGTTGGA-3′
	Reverse: 5′- GCCAAGCACAATTTGCCGTA-3′
Dctn3	Forward: 5′- ACTGAGGAGTCCAAGGCTCT-3′
	Reverse: 5′- TCCTCTGCTGGCTTCACTTG-3′
Fmn2	Forward: 5′-CCTGGGCTGCAGTTAGTCAA-3′
	Reverse: 5′- TGTTTCAGCATCCTCGAGCC-3′
Arl3	Forward: 5′-GCTGGCAAGACCACTCTTCT-3′
	Reverse: 5′-CCTCTGTCCACCAATGTCCC-3′

Plk3	Forward: 5′-CTACGAGGCCACTGACACAG-3′
	Reverse: 5′-CTCGGTGCAGCTCAATCTCA-3′
Jtb	Forward: 5′-AACGGGGACGTCCTGATAGA-3′
	Reverse: 5′-GCACTTACTCTGCAGCCCTC-3′
β-actin	Forward: 5′-GGGAGAAATGGTGGGCG-3′
	Reverse: 5′-GCCAGTCTGGGATCGTCATC-3′

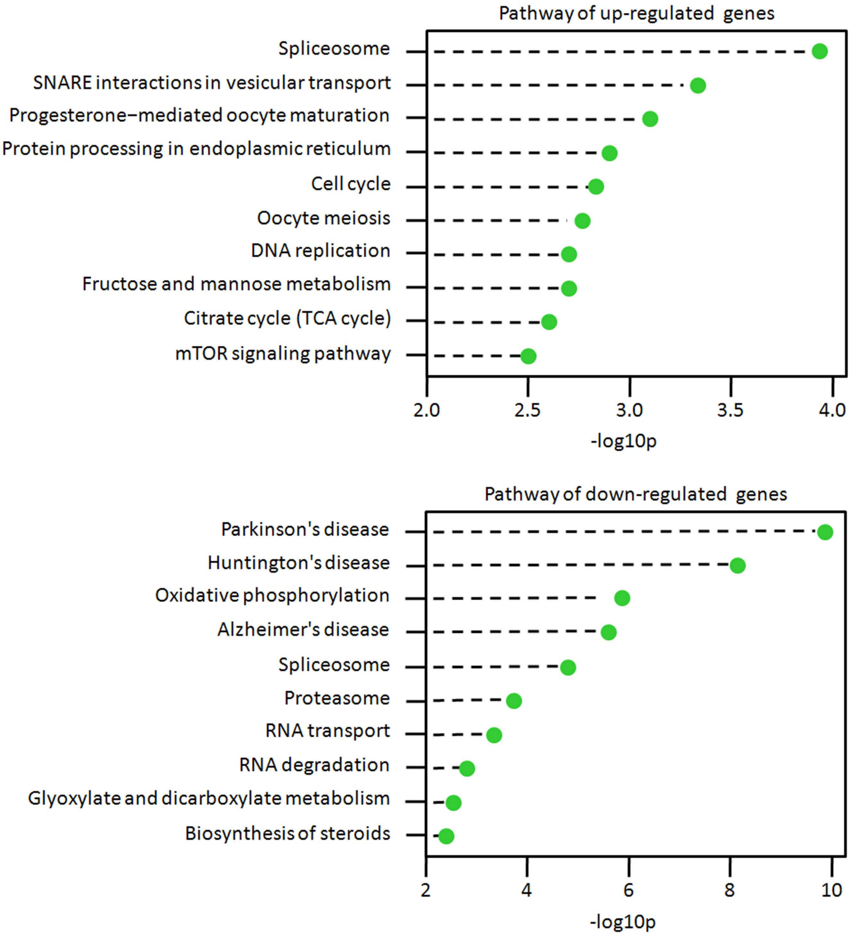

Pathway of up-regulated genes
Spliceosome
SNARE interactions in vesicular transport
Progesterone−mediated oocyte maturation
Protein processing in endoplasmic reticulum
Cell cycle
Oocyte meiosis
DNA replication
Fructose and mannose metabolism
Citrate cycle (TCA cycle)
mTOR signaling pathway
2.0
2.5
3.0
3.5
4.0
-log10p
Pathway of down-regulated genes
Parkinson's disease
Huntington's disease
Oxidative phosphorylation
Alzheimer's disease
Spliceosome
Proteasome
RNA transport
RNA degradation
Glyoxylate and dicarboxylate metabolism
Biosynthesis of steroids
2
4
6
8
10
-log10p

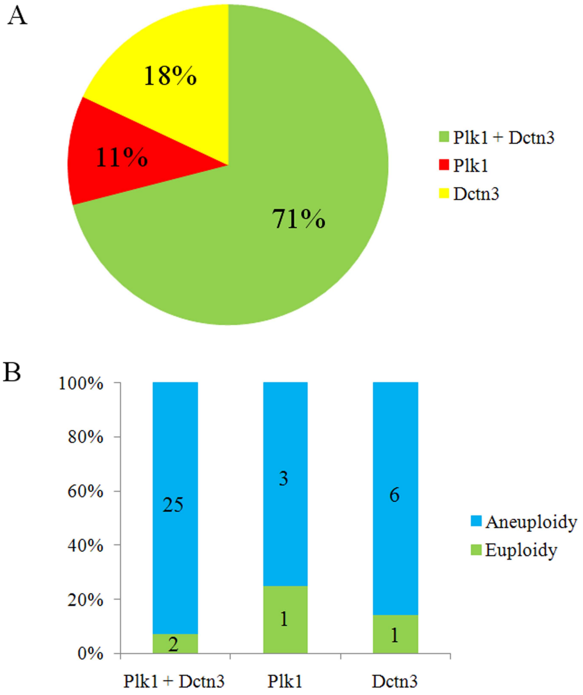

A
18%
11%
71%
Plk1 + Dctn3
Plk1
Dctn3
B
100%
80%
60%
40%
20%
0%
25
2
3
1
6
1
Aneuploidy
Euploidy
Plk1 + Dctn3
Plk1
Dctn3

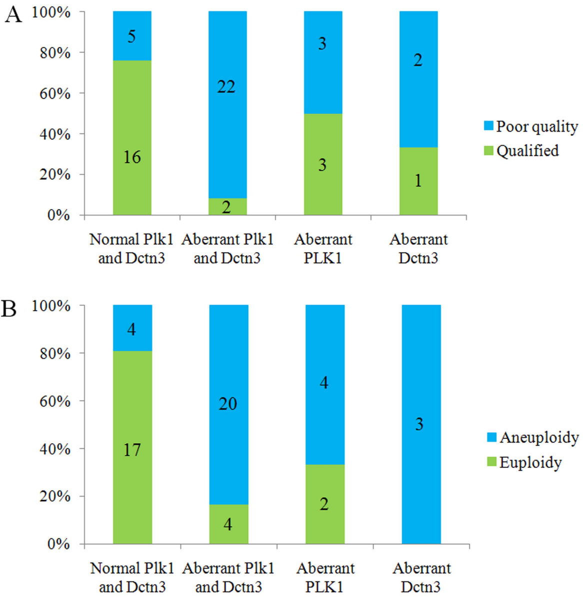
A
100%
80%
60%
40%
20%
0%
5
16
22
2
3
3
2
1
Poor quality
Qualified
Normal Plk1 and Dctn3
Aberrant Plk1 and Dctn3
Aberrant PLK1
Aberrant Dctn3
B
100%
80%
60%
40%
20%
0%
4
17
20
4
4
2
3
Aneuploidy
Euploidy
Normal Plk1 and Dctn3
Aberrant Plk1 and Dctn3
Aberrant PLK1
Aberrant Dctn3

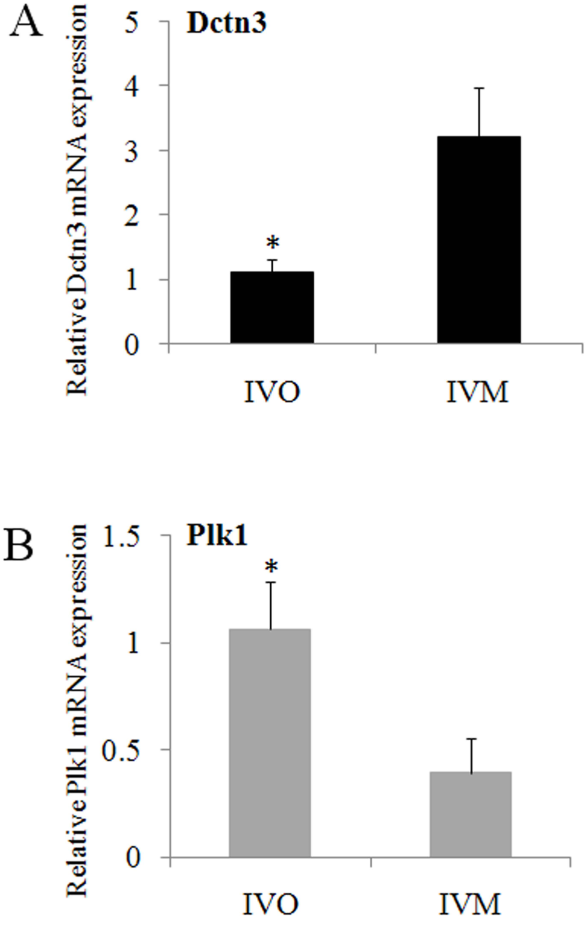
A
Dctn3
Relative Dctn3 mRNA expression
5
4
3
2
1
0
*
IVO
IVM
B
Plk1
Relative Plk1 mRNA expression
1.5
1
0.5
0
*
IVO
IVM

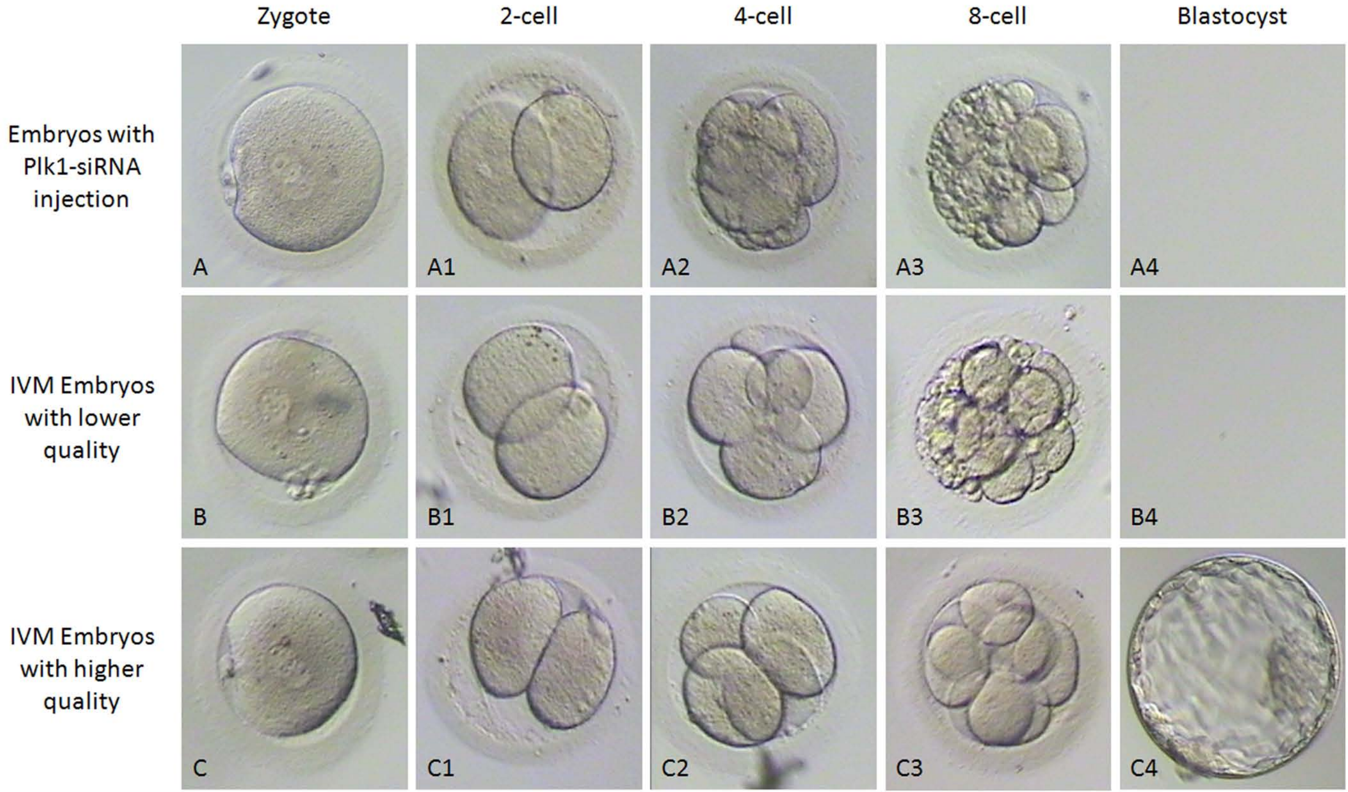
Zygote
2-cell
4-cell
8-cell
Blastocyst
Embryos with Plk1-siRNA injection
IVM Embryos with lower quality
IVM Embryos with higher quality
A
A1
A2
A3
A4
B
B1
B2
B3
B4
C
C1
C2
C3
C4
